# Impact of recombinant expression in *Komagataella phaffii* on the allergenic properties of the peanut allergen Ara h 2

**DOI:** 10.3389/fimmu.2025.1713823

**Published:** 2025-12-18

**Authors:** Andrea Wangorsch, Annette Jamin, Sonja Wolfheimer, Melanie Albrecht, Stefan Vieths, Jonas Lidholm, Stephan Scheurer

**Affiliations:** 1 Molecular Allergology, Paul-Ehrlich-Institut, Langen, Germany; 2 ThermoFisher Scientific, Uppsala, Sweden

**Keywords:** peanut allergy, 2S albumin, Ara h 2 allergen, IgE - binding, recombinant expression, Kex2 endoprotease, *Komagataella phaffii* (*P. pastoris*)

## Abstract

**Introduction:**

Recombinant allergens are an important diagnostic tool for determining the IgE-sensitization profile of patients, assessing the risk of symptom severity and potential clinical cross-reactivity. In this context, the use of different host cells for recombinant expression must be evaluated in terms of IgE reactivity and diagnostic value. Therefore, recombinant Ara h 2, the major peanut allergen, was produced in yeast, structurally characterized and investigated in respect to IgE-binding and allergenic properties.

**Methods:**

Ara h 2 was produced as recombinant protein using yeast and *E. coli* expression systems. Purified proteins were assessed using SDS-PAGE (reducing and non-reducing conditions), CD-spectroscopy and IgE-reactivity.

**Results:**

Recombinant Ara h 2^wt^ expressed in yeast resulted in an additional predominant band of approximately 12 kDa upon DTT treatment. In contrast, the molecular mass of rAra h 2 expressed in *E. coli* (Ara h 2*^E.coli^*) remained unaffected by reduction. Analysis of rAra h 2^wt^ confirmed the presence of two Ara h 2-derived peptides, one with the expected N-terminus and the other with an N-terminal glycine residue. *In silico* analysis revealed the presence of a Kex2 cleavage site (R_58_R_59_*G_60_). To test whether Kex2 cleavage affects an IgE-epitope, mutagenesis of this cleavage site from R_58_ to E_58_ (Ara h 2^mut^) was performed. DTT treatment of rAra h 2^mut^ purified from yeast showed that no cleavage of the protein had occurred. No effect on IgE binding could be observed as all rAra h 2 preparations showed IgE-reactivity. Cross-linking of human serum IgE and monoclonal human Ara h 2-specific IgE antibodies showed comparable mediator release in response to Ara h 2^wt^ and rAra h 2^mut^. However, utilizing a specific combination of human Ara h 2-specific IgE antibodies revealed slight epitope diversity between wildtype and mutated rAra h 2.

**Discussion:**

An endogenous protease, like Kex2 from the expression system, can affect the structural integrity of the target protein, leading to a slightly altered epitope structure. Even this finding has little or no impact for diagnosis, a suitable expression system and a detailed physico- and immunochemical characterization of recombinant allergens prior to their use as a diagnostic tool are of great importance.

## 1 Introduction

*In vitro* allergy diagnosis determining allergen specific IgE levels in serum or plasma of allergic patients is routinely used besides skin prick or allergen challenge tests. The allergen reagent used in these *in vitro* tests could be either complex protein extracts from allergenic source material or single purified allergens, being natural or recombinant proteins (1, 2). Determining allergen specific IgE against a panel of single allergenic molecules is referred to as component resolved diagnosis (CRD) (3). Using CRD, a patient’s IgE reactivity profile allows for more accurate risk assessment of the severity of systemic reactions and persistence of allergy, the identification of serological or potential clinical cross-reactivity, the monitoring of ongoing allergen immunotherapy, or provides data on the prevalence of sensitization (1–3).

Specific IgE levels to certain single allergens are often used as marker to distinguish between a true allergy and asymptomatic sensitization (sensitized but tolerant). In addition, some allergens function as biomarkers to predict the risk of a severe allergic reaction, such as for peanut allergy where anaphylactic reactions are frequent (1, 4, 5).

According to oral food challenge (OFC) tests, peanut allergy is one of the most frequent food allergies, with a prevalence of up to 1.5% among the overall population, depending on the geographical region (5, 6). Especially in children it is one of the most common food allergies in Western countries, often associated with severe reactions such as anaphylaxis (7). One of the major allergens in peanut (*Arachis hypogaea*) is Ara h 2, a 2S albumin belonging to the conglutin protein family (8, 9). In peanut sensitized patients, up to 92% have specific IgE to Ara h 2 (10–12) and individuals allergic to this protein are at a higher risk than others of developing severe symptoms (3). Especially in children, Ara h 2 can be used as a predictive marker to diagnose peanut allergy in the majority of patients (11) and the determination of Ara h 2 specific IgE levels can help reduce the number of provocation tests required to reach a diagnostic conclusion (13).

Ara h 2, a protein consisting of 139 amino acids (AA) with an apparent molecular mass of 17 kDa, has a compact conformation with five α-helices stabilized by four disulfide bonds, which makes the allergen heat stable and resistant to proteolytic digestion (14–16). Due to its importance as a highly relevant allergen in peanut allergy diagnosis, Ara h 2 was in the focus of many studies, e.g. to define IgE epitopes (15, 17, 18) or to prepare hypoallergenic Ara h 2 variants for the potential utilization in allergen specific immunotherapy (19–21). Many of these studies use recombinant (r) Ara h 2, produced in different expression systems like bacteria (*E. coli*, *Lactococcus lactis)* or tobacco plants (*Nicotiana benthamiana*) (14, 22–24). Furthermore, Ara h 2 is used as a recombinant protein in many diagnostic test systems. Advantages of using recombinant expression systems are high yield and reproducibility and absolute purity from other allergens whereas allergens purified from natural source materials are at risk of containing impurities including additional allergens from the same source material. Moreover, recombinant allergens are more suitable for production under good manufacturing practice (GMP).

However, if recombinant proteins are used for IgE diagnosis, it needs to be ensured that the molecules show comparable IgE binding properties as their natural counterparts. In line with this, a disruption of IgE-binding epitopes, e.g. by incorrect disulfide bond formation throughout the manufacturing process of recombinant Ara h 2, could alter the IgE-binding capacity (15). The expression system used can have different effects on the recombinant protein produced. For commercial production, the yield of expressed protein is an important factor. Depending on the protein family, *E. coli* expression systems often reach their limits, if the protein is only produced in small quantities or in inclusion bodies, meaning that a modified bacterial or a yeast expression system may achieve better yields (14, 25). Furthermore, proteins expressed in bacterial systems do not contain post-translational modifications, whereas production in *Nicotiana benthamiana*, for example, leads to the expression of hydroxyproline, as described for Ara h 2, which is post-translationally modified in its natural form (24). The same is described for Ara h 6, also a 2S albumin from peanut, where expression of the recombinant protein in yeast, but not in bacteria, preserves allergic effector function (26). Therefore, this study aimed to compare yeast and *E. coli* expression systems for preparation of recombinant Ara h 2 with respect to the integrity of the molecular structure and IgE binding capacity.

## 2 Materials and methods

### 2.1 Patient’s sera

Plasma from seven peanut sensitized subjects were purchased from AbBaltis (Sittingbourne, United Kingdom) and PlasmaLab (Everett, USA). Companies state: Our products are carefully sourced, ethically obtained, and designed to support the needs of IVD manufacturers, researchers, and laboratories worldwide (https://www.abbaltis.com/products); PlasmaLab is licensed by the U.S. Food and Drug Administration (FDA). All applicable FDA, Good Manufacturing Practices, HIPAA, and Clinical Laboratory Improvement Act regulations are strictly followed (https://plasmalab.com/faq/). Peanut and Ara h 2 specific IgE concentrations of the samples were determined using the ImmunoCAP™ system (Thermo Fisher Scientific, Dreieich, Germany) and are shown in **Supplementary Table S1**.

### 2.2 Production of recombinant Ara h 2

**Figure 1:**
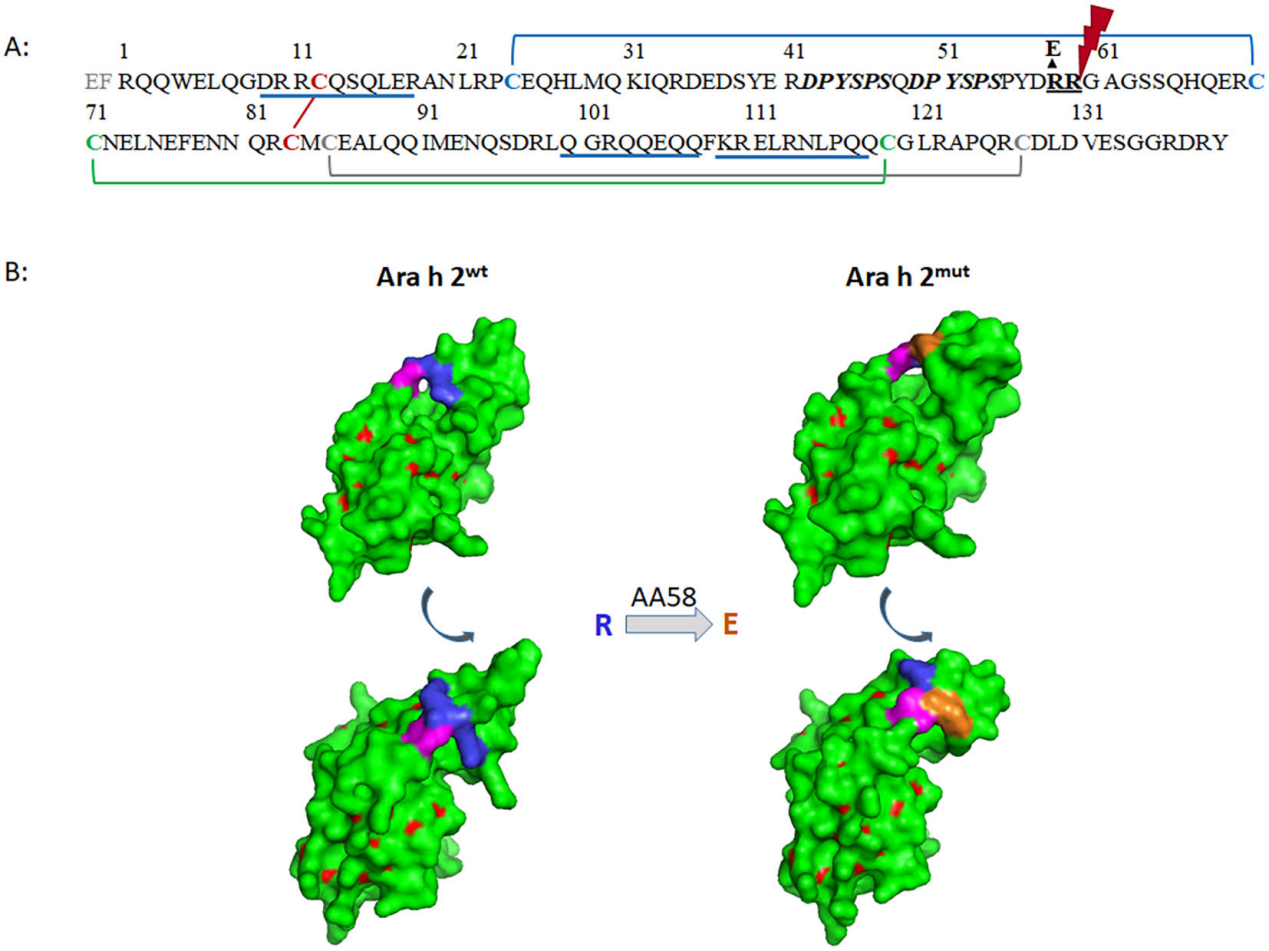
Sequence of Ara h 2.0101 without signal peptide, as expressed in yeast and *E coli*. **(A)** Eight cysteines forming the 4 disulfide bonds are shown in bold and respective linkages are illustrated with colored lines; the repetitive linear IgE binding epitope (AA_42–54_) is shown in italic; three sequential epitopes are underlined in blue; the RR motif at position 58/59 (bold underlined) serves as recognition sequence for Kex2 cleavage of which R at position 58 was changed to E; **(B)** 3D model with surface structure of Ara h 2^wt^ and Ara h 2^mut^, green: loop region, red: helix, blue: AA_58/59_ (RR), magenta: AA_60/61_ (GA), orange: mutated AA_58_ R58E.

The DNA sequence of the isoform Ara h 2.0101 (acc. no. ACN62248), hereinafter referred to as Ara h 2, was used as template to generate either *Komagataella (K.) phaffii* (Synonym: *Pichia pastoris*) (Ara h 2^wt^) or *E. coli* (Ara h 2*^E.coli^*) codon optimized synthetic genes (Eurofins Genomics, Ebersberg, Germany). Furthermore, one codon optimized gene intended for expression in *K. phaffii* was generated with a point mutation at amino acid (AA) position 58 introducing the exchange of arginine (R) to glutamic acid (E), resulting in the protein Ara h 2^mut^ (**Figure 1A**). The genes were cloned into the respective expression systems: (A) pET32a vector (Merck, Darmstadt, Germany) including thioredoxin (Trx)-Tag, His-Tag and a PreScission protease cleavage site, served for expression in *E. coli* Origami™ 2(DE3) cells (Merck, Darmstadt, Germany) as described by Lehmann et al. (14); (B) pPICZαA vector system (Thermo Fisher Scientific) was used for expression in *K. phaffii* X-33 cells (Thermo Fisher Scientific) as described by Lauer et al. (27) with slight modifications: When the pre-culture reached log phase growth (OD_600_ = 2-6), the cells were harvested by centrifugation and re-suspended in 200 ml BMMY medium (100 µg/ml Zeocin) to start the methanol utilizing expression. Usually, after five days of methanol (MeOH) feeding (reaching 0.1 to 0.5% MeOH final concentration), the culture supernatant, containing rAra h 2, was harvested by centrifugation and dialyzed against 20 mM Bis-Tris pH 6.2 buffer for purification. Two step chromatography using ion exchange (HiPrep Q HP, Cytiva, Munich, Germany) and gel filtration (HiPrep 26/60 Sephacryl S-100HR, Cytiva) with 20 mM MOPS, 150 mM NaCl pH 7.6 (or 20 mM Tris, 0.5 M NaCl, pH 8.0) as running buffer on an ÄKTA-FPLC (Cytiva) system was performed to obtain pure rAra h 2.

Protein purity and concentration were assessed using Coomassie (GelCode™ Blue Safe Protein Stain, Thermo Fisher Scientific) stained 14% sodium dodecylsulfate polyacrylamide gel electrophoresis (SDS-PAGE) and BCA assay (Pierce, Thermo Fisher Scientific), respectively. Structural integrity of all three rAra h 2 preparations was determined by circular dichroism (CD) -spectroscopy as described elsewhere (28).

### 2.3 N-terminal sequencing and mass spectrometric analysis of rAra h 2 expressed in yeast

To confirm the identity of yeast-expressed recombinant Ara h 2 and investigate potential internal cleavage sites, the purified protein was analyzed by N-terminal sequencing on an ABI Procise LC 492 instrument (Life Technologies, Carlsbad, CA, USA) and by mass spectrometry (MS) on an Orbitrap Fusion Tribrid instrument (Thermo Fisher Scientific, CA, USA). For sequence analysis by MS, the protein was reduced by DTT, alkylated with acrylamide and enzymatically cleaved with trypsin, chymotrypsin and Lys-C in separate reactions. MS data analysis was made on a combination of spectra obtained from the three digests, using PEAKS Studio software (Bioinformatics Solutions Inc). MS analysis of uncleaved and non-alkylated reduced protein was performed to determine its intact molecular mass and those of fragments occurring following reduction. Analysis of these spectra was performed using Xcalibur software (Thermo Fisher Scientific, CA, USA).

### 2.4 IgE immunoblotting

To investigate the IgE-binding capacity, rAra h 2 proteins were subjected to SDS-PAGE (16%, 1 µg/cm with 30 mM DTT) and transferred to 0.2 µm nitrocellulose membrane (Amersham Protran, Thermo Fisher Scientific) via semi dry blotting. The membrane was cut into strips and IgE detection was performed as described elsewhere (29), except using the plasma of Ara h 2 sensitized patients at a 1:10 dilution and Ara h 2 specific human monoclonal IgE antibodies (AB) at a concentration of 0.5 µg per strip. According to the manufacturer (Mabylon AG, Schlieren, Switzerland) the customized production of Ara h 2 specific human monoclonal antibodies was performed as described in Paolucci et al. (30) with the exception that for this study, antibody variable regions were cloned into human IgE expression vectors. Information about the epitope-specificity of the monoclonal antibodies were not disclosed by the manufacturer.

### 2.5 IgE-cross reactivity testing

To compare the IgE binding capacities of different rAra h 2 preparations, a competitive IgE binding test by enzyme linked immunosorbent assay (ELISA) was performed using plasma of Ara h 2 sensitized patients or Ara h 2 specific human monoclonal IgE ABs. High bind ELISA plates (Sarstedt, Nümbrecht, Germany) were coated with rAra h 2 (1 µg/ml for plasma or 0.2 µg/ml for AB) in coating buffer (50 mM sodium-carbonate pH 9.6) over night at 4°C. After blocking with phosphate-buffered saline (PBS) containing 0.5% Tween (PBS-T) and 2% bovine serum albumin (BSA, Thermo Scientific) for 2 h at room temperature (RT), an overnight incubation with plasma or AB mixed with different concentrations of the rAra h 2 preparations was carried out as previously described (31). Plasma and AB were diluted to reach an OD_450nm_ around 0.5 (P4 1:30, P7 1:250, P10 1:100, P11 1:60, P12 1:500, P14 1:400, ABs #1-3–20 ng/ml, AB #4–160 ng/ml). Dilution series (1:5) of inhibitors started at 25 µg/ml. BSA and major birch pollen allergen rBet v 1, produced as described by Heinl et al. (32), served as negative controls.

### 2.6 Mediator release assay

Allergenic potency of the rAra h 2 preparations was assessed using the humanized rat basophil leukemia cell (huRBL-2H3) assay as described previously (33). Plasma was diluted 1:20 and cross-linking with rAra h 2 was done using serial dilution (1:10) with a starting concentration of 1 µg/ml. Besides human plasma samples, Ara h 2 specific human IgE Abs as described in section 2.5 (customized production by Mabylon) were used for passive sensitization of huRBL-2H3 cells. To enable IgE-mediated cross-linking by Ara h 2, a mixture of two monoclonal ABs was used. The ABs were applied at a concentration of 0.5 µg/ml each, so that the final antibody concentration of the mixture was 1 µg/ml. Starting concentration (1 µg/ml) and serial dilution (1:10) of rAra h 2 samples, were as described above.

### 2.7 Structural modeling of Ara h 2^wt^ and Ara h 2^mut^

The 3D model of Ara h 2^mut^ was generated using SWISS-MODEL (34, 35) with Ara h 2.0101 (PDB: 8SJ6) as template and PyMOL Molecular Graphics System, Version 1.5.0.4 Schrödinger, LLC as modelling program. Alignment of AA sequences of different 2S albumins was performed using Clustal Omega and T-coffee (36) sequence analysis tools.

## 3 Results

### 3.1 Ara h 2 expressed in yeast is cleaved into two fragments

**Figure 2:**
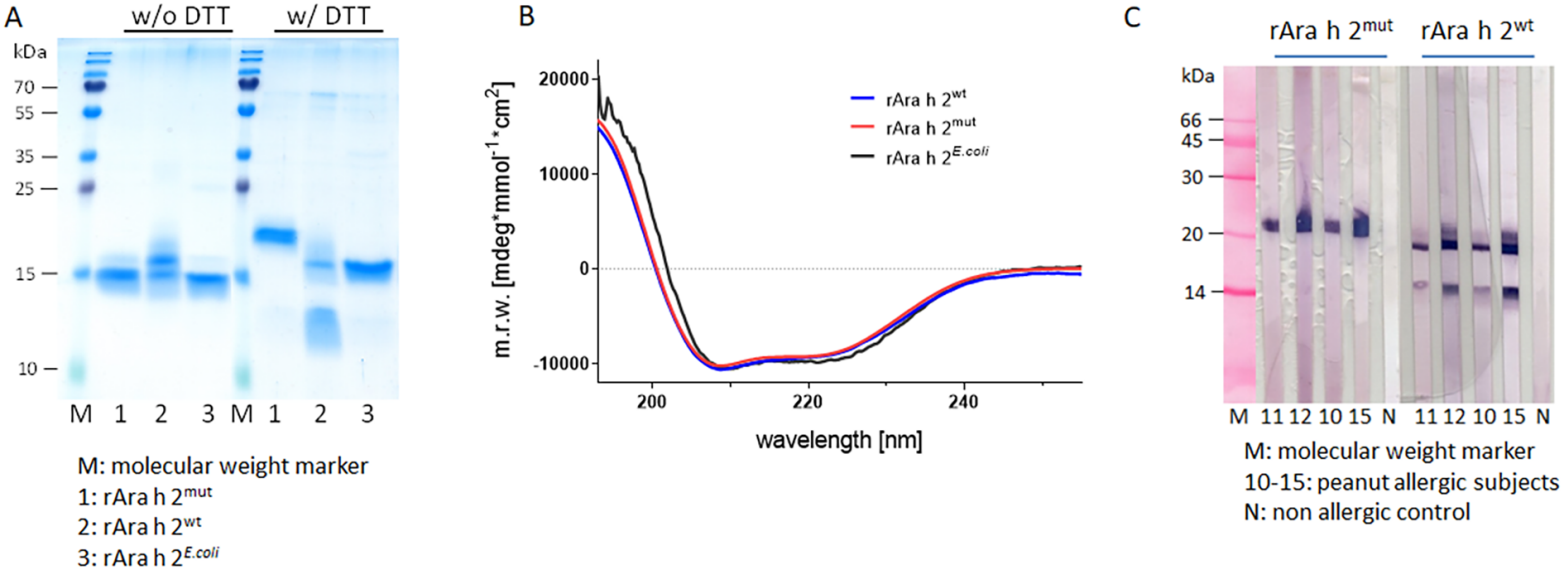
Yeast derived Ara h 2 is detectable as two protein bands under reducing conditions, but shows intact secondary structure and IgE reactivity. **(A)** Coomassie staining of Ara h 2 preparations under non-reducing (w/o DTT) and reducing (w/DTT) conditions in SDS-PAGE, 1: Ara h 2^mut^, 2: rAra h 2^wt^, 3: rAra h 2*^E.coli^*, M: molecular weight marker; **(B)** secondary structure elements of rAra h 2 preparations determined by CD-spectroscopy; **(C)** IgE reactivity of rAra h 2^wt^ and rAra h 2^mut^ under reducing (w/DTT) conditions in immunoblot using plasma of four Ara h 2 sensitized subjects (10–12, 15) and a non-allergic control (N), M: molecular weight marker.

In this study, the pPICZαA/*K. phaffii* yeast expression system was used for the generation of rAra h 2^wt^, which led to a yield of around 10 mg rAra h 2 per liter cell culture. Characterization of purified rAra h 2^wt^ using Coomassie stained SDS-PAGE showed two distinct bands of around 15 and 17 kDa under non-reducing conditions (**Figure 2A** w/o DTT, lane 2). However, under reducing conditions two proteins of approximately 12 kDa and 17 kDa can be visualized (**Figure 2A** w/DTT, lane 2). In silico analysis showed that Ara h 2^wt^ contains a potential Kex2 cleavage site consisting of the dipeptide RR at AA positions 58-59 (37). In general, the Kex2 endoprotease, which is endogenously produced during methanol induced protein expression in yeast, serves to cleave and remove the α-factor added to the yeast expressed protein for secretion to the culture medium.

Analysis of the DTT-treated protein (non-separated Ara h 2^wt^ fragments) revealed two different sequences determined by N-terminal sequencing, and the presence of the predicted fragments was confirmed by determination of the intact mass (7539.500 Da (N-terminal), 10203.651 Da (C-terminal), 17725,151 Da (full-length)). N-terminal sequencing confirmed the correct translation of Ara h 2^wt^ in the yeast system with an AA-sequence of *EF*R_1_QQ for the N-terminal start of the full-length protein, with the amino acids E_-2_F_-1_ (**Figure 1a**) resulting from the use of an EcoRI (GAATTC) restriction site. Additionally, a G_60_AGS amino acid sequence was determined, supporting the hypothesis that Kex2 cleaved the protein after R_58_R_59_ (**Figure 1A**). The experimental data fit with in silico analysis of Ara h 2 cleaved by Kex2, resulting in two peptides with similar masses of theoretically 7.3 kDa (AA_1-59_) and 9.4 kDa (AA_60-139_) which after DTT treatment appear as a smear at an apparent molecular mass of around 11–13 kDa in Coomassie staining (**Figure 2A** w/DTT, lane 2). Since only bands above 15 kDa could be detected under non-reducing conditions in Coomassie stained SDS-PAGE, the two polypeptide chains appear to be stabilized by internal disulfide bridges (**Figure 2A** w/o DTT, lane 2). To confirm the cleavage site present in rAra h 2^wt^, a point mutation at AA position 58 was introduced to the predicted Kex2 cleavage site by exchanging arginine (R_58_) to glutamic acid (E_58_), resulting in the protein Ara h 2^mut^. Modelling the 3D structure of Ara h 2 showed that the replacement of the amino acid at position 58 by glutamic acid probably seems to have the least effect on the protein structure compared to other possible mutations (**Figure 1B**, **Supplementary Figure S1**). The *K. phaffii* expression of Ara h 2^mut^ resulted in a yield of 4 mg purified protein per liter of culture.

For comparison with the proteins produced in yeast, Ara h 2 was expressed in *E. coli* according to the protocol described by Lehmann et al. (14) which led to a final yield of rAra h 2*^E.coli^* of 1.5 mg per liter of culture after purification and removal of the thioredoxin- and 6x His-tag via PreScission protease cleavage. SDS-PAGE analysis using Coomassie staining showed that both rAra h 2*^E.coli^* and Ara h 2^mut^ formed dominant single protein bands of around 15 kDa (**Figure 2A** w/o DTT, lanes 1 and 3). Following reduction with DTT, both rAra h 2^mut^ and Ara h 2*^E.coli^* still appeared as one distinct band but with slightly increased apparent molecular masses of around 20 kDa and 17 kDa, respectively (**Figure 2A** w/DTT, lanes 1 and 3).

### 3.2 Ara h 2 preparations show intact secondary structure and IgE reactivity

To check if the expression system or the mutation of Ara h 2 had an impact on the structural integrity of the allergen, CD-spectroscopy was performed. According to **Figure 2B** all Ara h 2 preparations showed intact secondary structure. Both yeast-derived proteins were nearly identical in their curve shape. rAra h 2*^E.coli^* had a slightly different x-axis passage at 202 nm compared to around 200 nm for the yeast derived proteins. However, the two minima indicate a similarly high α-helical content across all samples tested (**Figure 2B**).

To investigate if the point mutation in rAra h 2^mut^ had affected the IgE binding capacity of the protein, it was analysed by IgE-immunoblotting alongside rAra h 2^wt^ for comparison. The analysis was performed under reducing conditions to assess the IgE-binding of both fragments of Ara h 2^wt^. Using plasma of four Ara h 2 sensitized patients, both yeast-derived preparations displayed IgE-reactivity, and even the fragments of Ara h 2^wt^ showed IgE binding (**Figure 2C**).

When checking the IgE reactivity of the three rAra h 2 preparations using human monoclonal IgE antibodies, AB #4 shows only weak reactivity under non-reducing conditions, whereas under reducing conditions the IgE binding is comparable with ABs #1-3 (**Supplementary Figure S3**).

### 3.3 The IgE-binding capacity of differently expressed Ara h 2 molecules are comparable

**Figure 3:**
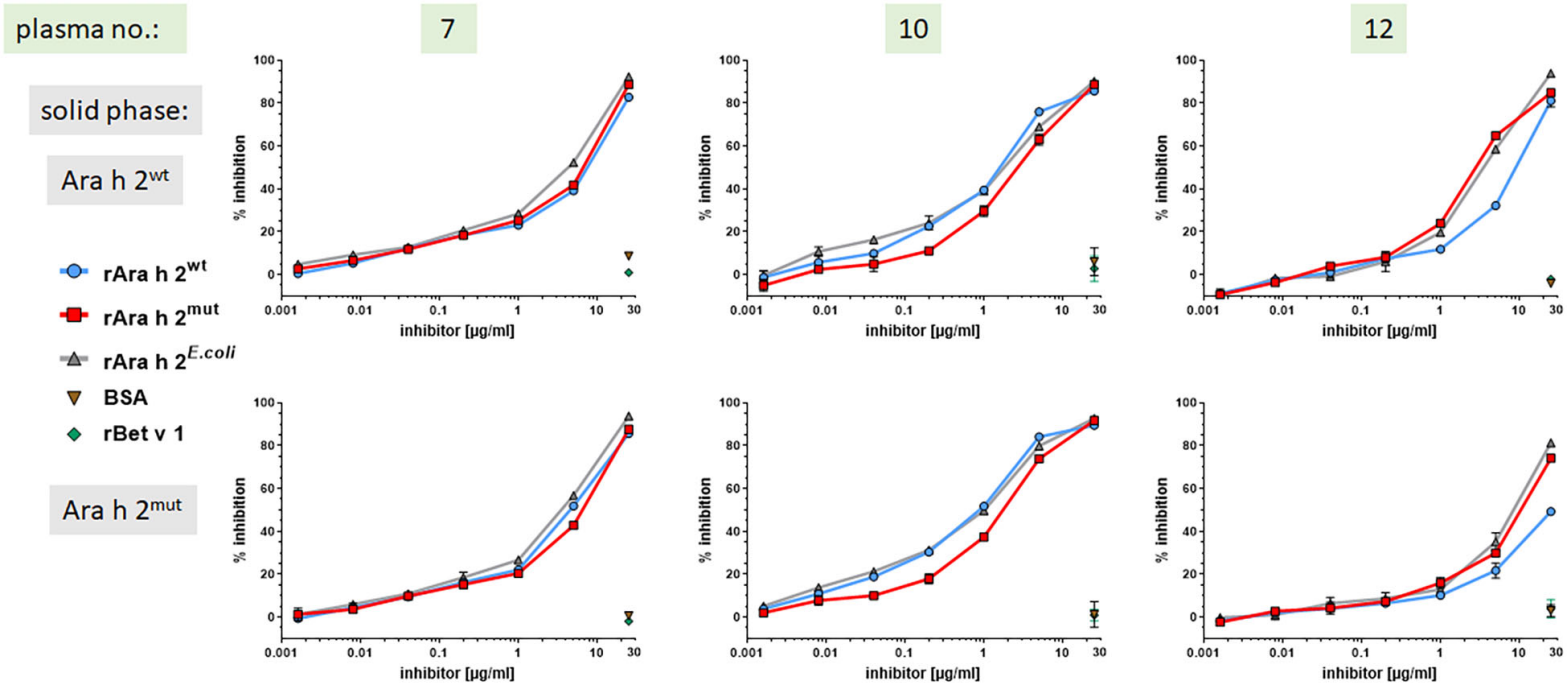
All Ara h 2 preparations show similar IgE-binding capacity using plasma of Ara h 2 sensitized subjects. Competitive ELISA inhibition was performed using Ara h 2^wt^ or Ara h 2^mut^ on the solid phase and inhibition of IgE binding by addition of the respective Ara h 2 preparation (25 µg/ml – 1.6 ng/ml) to plasma of three Ara h 2 sensitized patients (3/6 representative experiments are shown); BSA and rBet v 1 served as negative controls.

**Figure 4:**
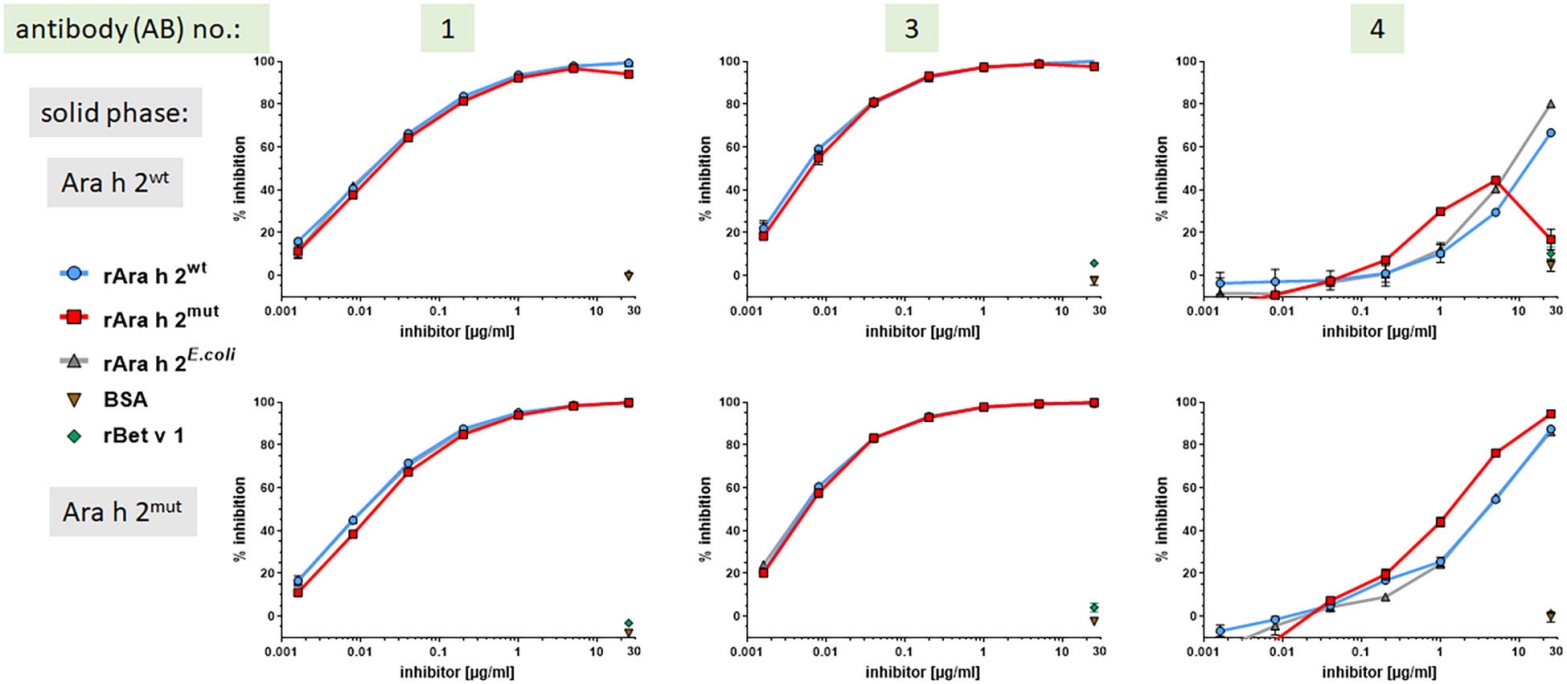
All Ara h 2 preparations show similar IgE-binding capacity to human Ara h 2 specific monoclonal IgE antibodies. Competitive ELISA inhibition was performed using Ara h 2^wt^ or Ara h 2^mut^ on the solid phase and inhibition of IgE binding by addition of the respective Ara h 2 preparation (25 µg/ml – 1.6 ng/ml) to three different Ara h 2 specific monoclonal IgE antibodies (3/4 representative experiments are shown).

To compare the IgE-binding capacities of rAra h 2^wt^ and rAra h 2^mut^ in more detail, IgE cross-inhibition experiments using ELISA were performed. To this end, the binding of IgE from Ara h 2 sensitized subjects and human monoclonal Ara h 2 specific IgE antibodies was inhibited with different concentrations of the three rAra h 2 preparations. The results (**Figures 3**, **4**) showed that regardless of whether rAra h 2^wt^ or rAra h 2^mut^ was coupled to the solid phase, the inhibitory capacity of all Ara h 2 preparations within each patient sample (3 representative out of 6 patients shown) was very similar, (**Figure 3**). BSA and the irrelevant birch pollen allergen Bet v 1 served as negative controls and did not show any inhibition of IgE binding to Ara h 2.

In regard to using human monoclonal IgE ABs #1 to #3 (AB #2 not shown) they performed almost identical, whereas AB #4 showed a different curve shape (**Figure 4**). Therefore, AB #4 appears to bind to a different epitope and/or has a different affinity, which was consistent with the finding that a higher concentration of inhibitor was required to achieve the same competitive activity (% inhibition) for AB #4 as for ABs #1 to #3.

### 3.4 All Ara h 2 preparations show the capacity to induce mediator release from effector cells

**Figure 5:**
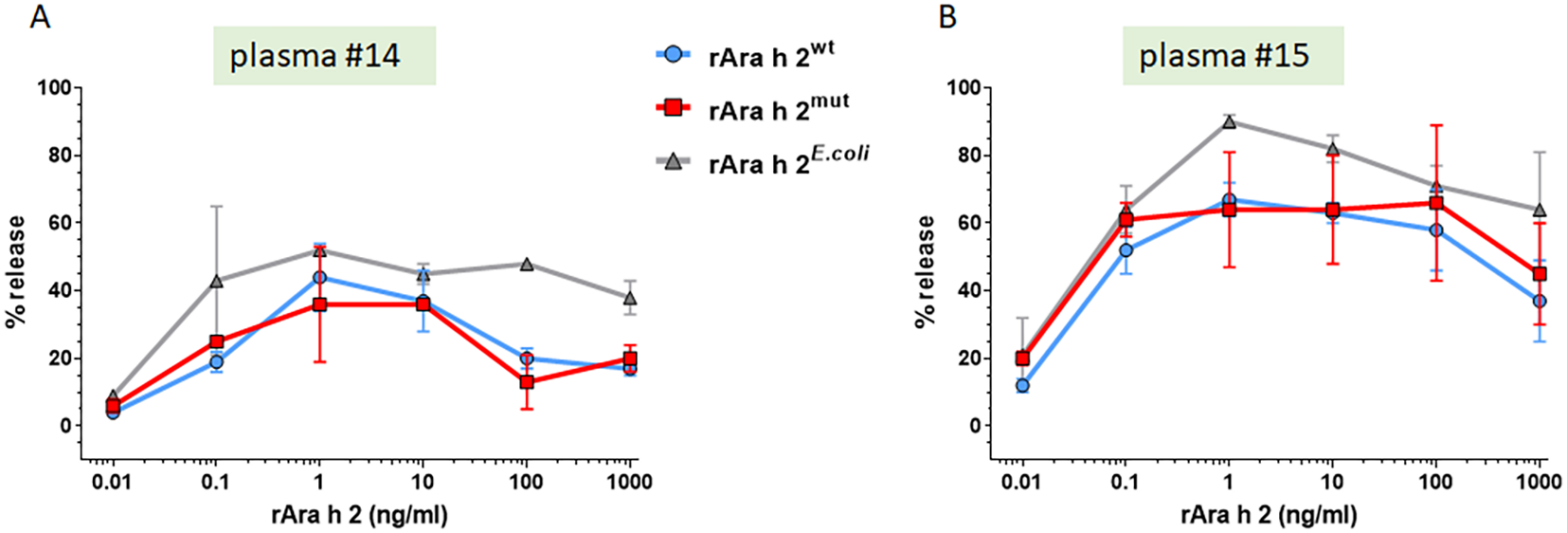
All Ara h 2 preparations are able to cross-link FcεR1-bound Ara h 2 specific IgE antibodies. The efficacy of Ara h 2 (0.01–1000 ng/ml) samples to induce mediator release was determined by the humanized RBL assay using different plasma samples (n=2); **(A)** plasma number 14; **(B)** plasma number 15.

Using the humanized RBL assay, the various Ara h 2 preparations were tested for their efficacy in inducing IgE-mediated mediator release. In both patients tested, Ara h 2^mut^ as well as Ara h 2^wt^ and Ara h 2*^E.coli^*, showed a clear ability to trigger the release of ß-hexosaminidase (**Figure 5**). Both yeast-derived preparations (Ara h 2^mut^ and Ara h 2^wt^) showed an almost identical dose-dependent curve shape with regard to the threshold value for triggering maximum release. A slightly higher mediator release capacity observed for Ara h 2*^E.coli^* was not statistically significant.

**Figure 6:**
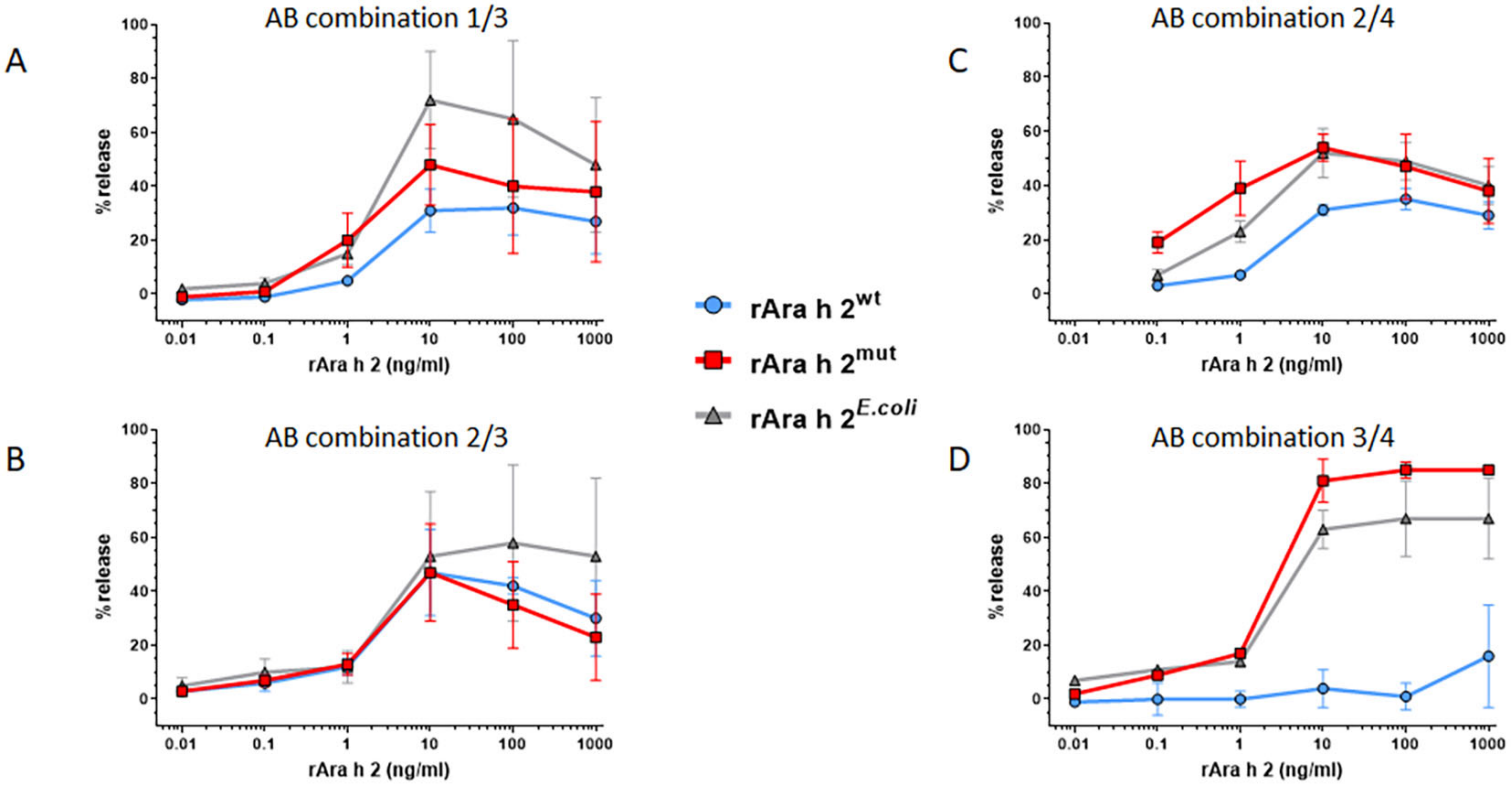
Human monoclonal Ara h 2 specific IgE antibodies show different properties in the potency assay. The potency of Ara h 2 (0.01–1000 ng/ml) samples to induce mediator release was determined with the humanized RBL assay using human IgE ABs (n=4) in different combinations; **(A)** mix of AB 1 + 3, **(B)** mix of AB 2 + 3, **(C)** mix of AB 2 + 4, **(D)** mix of AB 3 + 4.

Furthermore, all three Ara h 2 preparations were able to trigger mediator release when using different combinations of human-derived Ara h 2 specific IgE monoclonal antibodies (**Figure 6**). Compared to Ara h 2^mut^ and Ara h 2*^E.coli^*, Ara h 2^wt^ tended to show a lower maximum mediator release capacity in this experiment. Comparing the two preparations from *K. phaffii*, this finding was particularly evident with the combination ABs #3 and #4, in which no activation of the basophils with Ara h 2^wt^ was observed (**Figure 6D**).

## 4 Discussion

The determination of allergen specific IgE in sera of patients is a widespread test procedure for allergy diagnosis. Especially, for the so-called component resolved diagnostics, single purified allergens from an allergenic source are utilized to investigate the sensitization profile of the patients. Since purification of natural allergens from the respective allergenic sources often does not yield sufficient quantities or purity, generation of recombinant allergens is a frequently used alternative option. Expression systems employing *E. coli* or yeast cells are typically used. In some cases, plant (e.g. *Nicotiana* species) or insect cells are applied. In order to ensure the fitness for diagnostic use of such recombinantly expressed allergens, extensive characterization of the recombinant proteins in regard of structural integrity, stability, post-translational modifications, IgE-binding capacity and biological activity has to be performed.

Sensitization towards the major peanut allergen Ara h 2 serves as diagnostic marker in severity assessment of peanut allergy (5, 11). Since the purification of natural Ara h 2 is laborious and frequently accompanied by contamination of Ara h 6 (38) and potentially other peanut proteins, recombinant expression is a suitable strategy to circumvent these problems. Therefore, in the present study we used the yeast *K. phaffii* system to express structurally intact rAra h 2, a 2S albumin characterized by four intramolecular disulfide bonds.

When analyzing rAra h 2 from *K. phaffii* in SDS-PAGE under non-reducing conditions, the protein was detectable as two protein bands with apparent molecular masses of 15 and 17 kDa. Generally, this is described for natural Ara h 2 (39) and also for rAra h 2 produced in *Nicotiana benthamiana* (24) or for commercially available rAra h 2 expressed in *K. phaffii* (RP-AH2-1, InBio, Charlottesville, USA). However, the reason for the appearance of the two protein bands has not been clarified. We speculate that these two protein bands of rAra h 2^wt^ are folding variants or proteins with different post-translational modifications which can be formed in eukaryotic host cells but not in *E. coli*. In line with this, Ara h 2*^E.coli^* does not show the pronounced double band. Noteworthy, the commercially available rAra h 2 generated in yeast exhibits the same protein pattern, even though it is a different isoform (Ara h 2.0201) which has a short insertion of 12 AA at position 50 but shares 98.6% AA identity outside of that insertion and also contains the potential internal Kex2 cleavage site.

Surprisingly, rAra h 2^wt^ from *K. phaffii* exhibited two protein bands, one with a molecular size of 17 kDa and an additional fragment of around 12 kDa, when analyzed by SDS-PAGE under reducing conditions. The fact that the dissociation of the rAra h 2^wt^ peptides can only be observed under reducing conditions shows that the choice of SDS-PAGE conditions has a significant influence on the characterization and evaluation of recombinant proteins. To the best of our knowledge, this is the first study, where rAra h 2 produced in yeast was characterized on the molecular level, showing that the expression system is responsible for a cleavage of the target protein.

More detailed analysis led to the assumption that the endogenously produced yeast Kex2 protease partly cleaves the recombinant produced Ara h 2^wt^ during *K. phaffii* expression, as Ara h 2 contains a potential Kex2 cleavage site RR at AA_58-59_ (37). This fact was corroborated by *in solution* MS-analysis of rAra h 2^wt^, which confirmed the N-terminal sequences of the two polypeptides. Furthermore, under non-reducing conditions, the protein shows the expected molecular size in SDS-PAGE. Typically the protein scaffold (3 dimensional structure) of 2S-albumins is stabilized by four disulfide bonds (40). Therefore, we hypothesize that these four internal disulfide bonds are able to stabilize the two polypeptides resulting from the Kex2 cleavage. Next, we considered whether the cleavage of Ara h 2 by Kex2 might affect IgE-binding epitopes which is relevant for diagnostic purposes. One prominent linear IgE epitope of Ara h 2 is the DPYSPS motif, which is repeated twice (Ara h 2.0101, **Figure 1A**) or three times (Ara h 2.0201) depending on the isoform (15, 24, 41). This epitope (AA_42–47_ and AA_49-54_, **Figure 1A**) is near the Kex2 cleavage site but should not be directly affected by the cleavage. However, Üzülmez et al. (24) could show that the hydroxylation of the proline residues plays a major role in IgE binding, which could explain the outcompeting of rAra h 2 by the natural (n) one in regard of IgE binding and basophil activation. Furthermore, three additional sequential IgE epitopes of Ara h 2 (**Figure 1A**, underlined in blue) were found by comparing the epitope and paratope repertoire of patients who underwent peanut oral immunotherapy (42). These three epitopes are not in the region of the Kex2 cleavage site (**Figure 1A**) and should even not be affected. The presence of such linear epitopes in both peptides of the cleaved Ara h 2 could explain the retained IgE reactivity of the polypeptides determined by IgE-immunoblot detection under reducing conditions (**Figure 2C**). One linear epitope found in Ara h 2.0201 (AA_60-74_) (43) is very similar to the sequence of Ara h 2.0101, but included the Kex2 cleavage site (AA_49-61_). Hence, one could speculate that reduced IgE binding might occur in patients where this epitope is a dominant target of Ara h 2 specific IgE. Since the recombinant Ara h 2^wt^ produced by yeast is cleaved into two proteins after DTT treatment, this is a substantial finding where the reduction of Ara h 2 has an impact on its molecular properties. Furthermore conformational epitopes are involved in IgE binding to Ara h 2, as unfolded (reduced and alkylated) rAra h 2 shows no or reduced IgE binding, depending on the peanut allergic individual (15, 44). Using chimeric proteins, Hazebrouck et al. (18) found one immunodominant conformational epitope in close proximity to the DPYSPS motif. Chen et al. (16) observed similar results when determining Ara h 2 binding mimotopes via a phage display library. As the Kex2 cleavage site is located within this region, the cleavage of Ara h 2 might have an impact on the IgE binding capacity.

To investigate experimentally whether Kex2 cleavage has an impact on the structural and allergenic properties of the protein, we applied two additional strategies for rAra h 2 expression. One was the mutation of the AA sequence at the Kex2 cleavage site by replacing arginine at position 58 with glutamic acid (R58E, Ara h 2^mut^). Based on in silico modelling of different potential AA exchanges at position 58 and 59 of the Ara h 2 sequence (**Supplementary Figure S1**), the substitution of R_58_ to E_58_ seemed to have the least impact on the structure of Ara h 2. The resulting protein (Ara h 2^mut^) expressed by the *K. phaffii* yeast system showed indeed nearly identical secondary structure elements as the unmutated protein (Ara h 2^wt^) as determined by CD-spectroscopy. Likewise, the CD-spectrum of Ara h 2*^E. coli^* produced using the *E. coli* thioredoxin system (14), showed very similar curve shapes, with only slightly different x-axis intersection and curve minima. Similar differences were also described for Ara h 6, another 2S-albumin from peanut, when recombinant expression in *E. coli* and yeast were compared (26).

With regard to Ara h 2^mut^, no additional bands in SDS-PAGE under reducing conditions were observed, indicating that this protein is not affected by the Kex2 protease during production. As outlined above, retained IgE-binding capacity and biological potency is of utmost importance in regard to the use of recombinant allergens for the purpose of IgE-based diagnostics. Both parameters were tested using IgE cross-inhibition and mediator release assays. Within these experiments using patient samples, no clear or significant differences between the three Ara h 2 preparations could be observed. However, the IgE binding of human Ara h 2 specific monoclonal IgE antibody #4 to Ara h 2^wt^ appeared to be partially impaired, indicating slight epitope variation between the Ara h 2 molecules. The fact, that in Immunoblot IgE-detection AB #4 shows a diminished reactivity under non-reducing conditions, but a comparable binding activity under reducing conditions, compared to ABs #1-3, could indicate that this antibody mainly recognizes linear epitopes (**Supplementary Figure S3**). This fact could explain the different performance in ELISA-inhibition and mediator release assay.

More important for diagnostic applications is that the different Ara h 2 preparations showed comparable IgE-binding capacity and allergenic potency when assessed with samples from of Ara h 2 sensitized patients. Based on inhibition and mediator release assays, we conclude that for Ara h 2, the expression system and, in particular, the cleavage have no significant impact on IgE binding properties. The protein conformation is probably stabilized under non-reducing conditions by the four disulfide bonds, which appeared to be correctly formed. Furthermore, many studies have shown that linear epitopes contribute to the IgE binding of Ara h 2, meaning that the IgE binding should be preserved even if the protein was cleaved into separate peptides or showed a partially disrupted tertiary structure.

An amino acid sequence comparison of 25 allergenic 2S albumins listed in the IUIS allergen database has shown that only Ara h 2 (both isoforms), Ara h 7 and Gly m 8 contain the presumed Kex2 cleavage site (**Supplementary Figure S2**). Therefore, potential Kex2 cleavage of yeast-produced 2S albumins so far only concerns peanut and soy. Nevertheless, possible impact of proteolytic cleavage on IgE-binding properties should always be kept in mind when evaluating expression systems for recombinant allergens.

In summary, for diagnostic purposes, where Ara h 2 is used exclusively for IgE-detection, the expression system used for production appears to play only a minor role. However, a precise and detailed analysis of the recombinant protein and its IgE binding capacity must be carried out before use. Especially for scientific purposes, where the intact structure is essential, such as epitope analysis, the expression system must be carefully selected.

## 5 Concluding remarks

For scientific questions, e.g. in-depth epitope analysis of Ara h 2, where structural changes might play a role, the choice of expression system should be considered, as this can have a significant impact on the results. In routine diagnostics, Ara h 2 produced by expression in yeast can likely be used without affecting the quality and sensitivity of the results as long as the proteins are used under non-reducing conditions.

## Data Availability

The raw data supporting the conclusions of this article will be made available by the authors, without undue reservation. Original N-terminal sequencing data are available at: https://doi.org/10.6084/m9.figshare.30868382.

## Supplementary material

Supplementary Table 1 Peanut (f13) and rAra h 2 (f423) specific IgE of plasma samples as determined using the ImmunoCAP System.

Supplementary Figure 1 3D model of Ara h 2^wt^ and different Ara h 2 mutations. green: loop region, red: helix region; blue: AA_58/59_ (RR), magenta: AA_60/61_ (GA), orange: mutated AA at position 58 or 59.

Supplementary Figure 2 AA-alignment of 25 allergenic 2S albumins listed in the IUIS allergen database. The region of the Kex2 cleavage site is marked by a box.

Supplementary Figure 3 IgE detection of rAra h 2^wt^, rAra h 2^R58E^ and rAra h 2*^E.coli^* using the four different human monoclonal Abs #1-4; M: molecular weight marker, C: secondary antibody control.
