# Supplementary figures and images for: Impact of recombinant expression in Komagataella phaffii on the allergenic properties of the peanut allergen Ara h 2
Source: Front Immunol. 2025 Dec 18;16:1713823. doi: 10.3389/fimmu.2025.1713823 (PMC12756488; doi:10.3389/fimmu.2025.1713823)

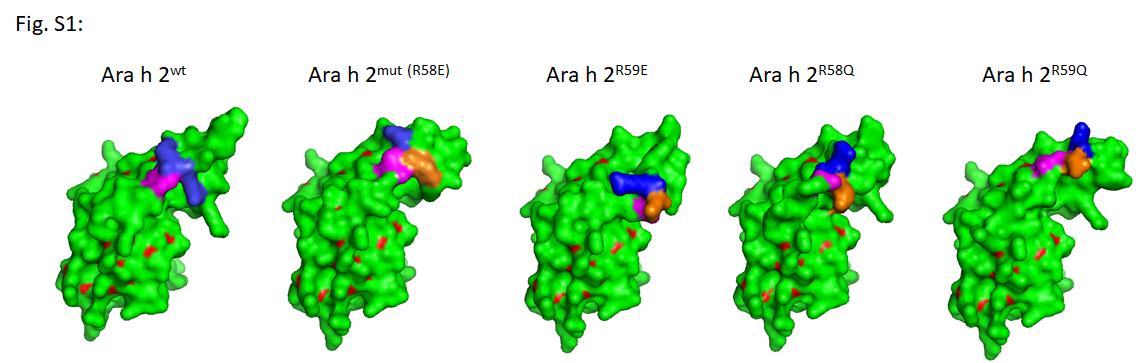

Supplement: Supplementary Figure 1 — 3D model of Ara h 2wt and different Ara h 2 mutations. green: loop region, red: helix region; blue: AA58/59 (RR), magenta: AA60/61 (GA), orange: mutated AA at position 58 or 59. [file Image1.tif]

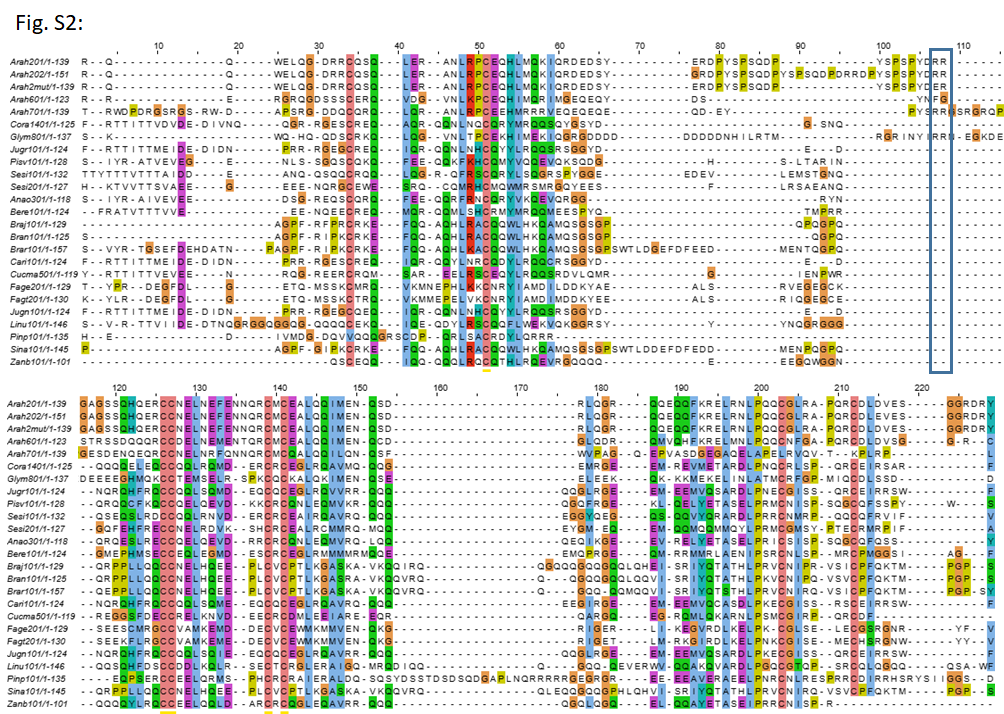

Supplement: Supplementary Figure 2 — AA-alignment of 25 allergenic 2S albumins listed in the IUIS allergen database. The region of the Kex2 cleavage site is marked by a box. [file Image2.tif]

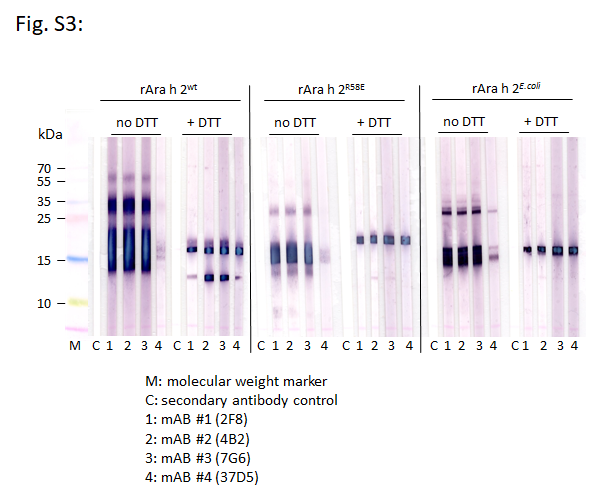

Supplement: Supplementary Figure 3 — IgE detection of rAra h 2wt, rAra h 2R58E and rAra h 2E.coli using the four different human monoclonal Abs #1-4; M: molecular weight marker, C: secondary antibody control. [file Image3.tif]
